# S150. DOPAMINE SYNTHESIS CAPACITY IN ANTIPSYCHOTIC NAïVE FIRST EPISODE PSYCHOTIC PATIENTS

**DOI:** 10.1093/schbul/sby018.937

**Published:** 2018-04-01

**Authors:** Anne Sigvard, Karen Tangmose, Kirsten Borup Bojesen, Kasper Jessen, Mette Ødegaard Nielsen, Dan Fuglø, Lars Thorbjørn Jensen, Egill Rostrup, Birte Glenthøj

**Affiliations:** 1 Center for Clinical Intervention and Neuropsychiatric Schizophrenia Research, Mental Health Centre Glostrup, University of Copenhagen; 2 Herlev Hospital, University of Copenhagen

## Abstract

**Background:**

Insufficient response to antipsychotics constitutes a challenge in the treatment of patients suffering from schizophrenia. Treatment resistances have been linked to a normal striatal dopamine system. We aim to stratify antipsychotic-naïve first-episode patients based on striatal dopamine synthesis capacity (DSC) measured with positron emission tomography (PET). We hypothesize that patients who respond to treatment have an increased DSC at baseline compared to non-responders and healthy controls (HC).

**Methods:**

The current data have been collected as a part of a multimodal first episode study. Patients are examined before and after 6 weeks treatment with flexible doses of Aripiprazole.

PET: Dynamic scans are performed in an integrated PET-CT scanner using the tracer 3,4-dihydroxy-6-[18F]fluoro-L-phenylalanine (18F-FDOPA). Duration of scanning is two times one hour, with half an hour break.

Arterial blood samples are collected during the scanning sessions and provide information on the ratio between intact 18F-FDOPA and the primary metabolite. The input functions used for this data analysis are image derived based on the largest cerebral vessels and metabolite corrected.

Regions of interest (ROIs) are manually drawn around the cerebellum and semi automatically around the basal ganglia. In this preliminary work, DSC values are based on Ki parameters obtained from slopes on Patlak plots.

**Results:**

DSC has been measured at baseline on 16 patients (mean age 22.6 years, 5 males) and 18 HC (mean age 21.7 years, 9 males), with no significant difference in age or gender between groups.

At baseline patients had a PANSS total score of 74 (SD 9.6) and GAF total score of 35 (SD 5.2).

No significant difference in Ki at baseline was shown between patients and HC. Clinical follow-up data was available on 12 patients. They received a mean Aripiprazole-dose of 9.3 (SD 3.9). Paired t-test at showed a significant effect of treatment with a follow up PANSS total of 55 (SD 11.9, p<0.001) and GAF total of 49 (SD 11.2, p=0.002).

Six patients were characterized as responders, and six as non-responders using the Nancy Andreasen remission criteria. There were no baseline differences in PANSS or GAF scores between responders and non-responders. Nor did the dose of Aripiprazole differ between these groups. There was however significant difference in the GAF total score at follow up (p =0.001), as GAF was 59 (SD 9.3) for responders and 39 (SD 5.3) for non-responders.

Mean Ki-values at baseline was 0.79 (SD 0.2) for non-responders, 0.88 (SD 0.2) for responders and 0.91 (SD 0.2) for HC. One way ANOVA showed no significant group difference.

**Discussion:**

Although not significant, we found a slightly lower Ki-value at baseline for non-responders compared to baseline Ki-values for responders and HC in these preliminary analyses. This was unexpected, but should be taken with precaution, as the results represent work in progress.

Inclusion of subjects and data-analyses is ongoing, and data analysis will be more extensive in spring 2018, especially regarding the methodology:

The current image derived input function suffers from partial volume effects (PVE). To account for PVE and other factors the image data will be co-registered with T1-weighted MRI data and normalized to standard space in order to use a standard anatomical atlas to help define the ROIs. Finally, as mentioned earlier, arterial samples are collected and we plan to use arterial input functions to correct for the complicated kinetics of the tracer. To improve the Ki estimation we will use the metabolite corrected arterial plasma curve as input function and compare these results with the current method.

